# Changes of DNA methylation are associated with changes in lung function during adolescence

**DOI:** 10.1186/s12931-020-01342-y

**Published:** 2020-04-07

**Authors:** Shadia Khan Sunny, Hongmei Zhang, Faisal I. Rezwan, Caroline L. Relton, A. John Henderson, Simon Kebede Merid, Erik Melén, Jenny Hallberg, S. Hasan Arshad, Susan Ewart, John W. Holloway

**Affiliations:** 1 Division of Epidemiology, Biostatistics, and Environmental Health, School of Public Health, University of Memphis, Memphis, TN 38152 USA; 2 School of Water, Energy and Environment, Cranfield University, Cranfield Bedfordshire, MK43 0AL England; 3 MRC Integrative Epidemiology Unit, University of Bristol, Bristol, BS8 2BN UK; 4 Population Health Sciences, University of Bristol, Bristol, BS8 2BN UK; 5 Department of Clinical Sciences and Education Södersjukhuset, Karolinska Institutet, Stockholm, Sweden; 6 Sachs’ Children’s Hospital, Stockholm, Sweden; 7 Clinical and Experimental Sciences, Faculty of Medicine, University of Southampton, Southampton, SO16 6YD UK; 8 The David Hide Asthma and Allergy Research Centre, St Mary’s Hospital, Parkhurst Road, Newport, Isle of Wight PO30 5TG UK; 9 Large Animal Clinical Sciences, Michigan State University, East Lansing, MI USA; 10 Human Development and Health, Faculty of Medicine, University of Southampton, Southampton, SO16 6YD UK

**Keywords:** Lung function, DNA methylation, Genome-wide, Adolescence, IOW cohort, ALSPAC, BAMSE

## Abstract

**Background:**

Adolescence is a significant period for the gender-dependent development of lung function. Prior studies have shown that DNA methylation (DNA-M) is associated with lung function and DNA-M at some cytosine-phosphate-guanine dinucleotide sites (CpGs) changes over time. This study examined whether changes of DNA-M at lung-function-related CpGs are associated with changes in lung function during adolescence for each gender, and if so, the biological significance of the detected CpGs.

**Methods:**

Genome-scale DNA-M was measured in peripheral blood samples at ages 10 (*n* = 330) and 18 years (*n* = 476) from the Isle of Wight (IOW) birth cohort in United Kingdom, using Illumina Infinium arrays (450 K and EPIC). Spirometry was conducted at both ages. A training and testing method was used to screen 402,714 CpGs for their potential associations with lung function. Linear regressions were applied to assess the association of changes in lung function with changes of DNA-M at those CpGs potentially related to lung function. Adolescence-related and personal and family-related confounders were included in the model. The analyses were stratified by gender. Multiple testing was adjusted by controlling false discovery rate of 0.05. Findings were further examined in two independent birth cohorts, the Avon Longitudinal Study of Children and Parents (ALSPAC) and the Children, Allergy, Milieu, Stockholm, Epidemiology (BAMSE) cohort. Pathway analyses were performed on genes to which the identified CpGs were mapped.

**Results:**

For females, 42 CpGs showed statistically significant associations with change in FEV_1_/FVC, but none for change in FEV_1_ or FVC. No CpGs were identified for males. In replication analyses, 16 and 21 of the 42 CpGs showed the same direction of associations among the females in the ALSPAC and BAMSE cohorts, respectively, with 11 CpGs overlapping across all the three cohorts. Through pathway analyses, significant biological processes were identified that have previously been related to lung function development.

**Conclusions:**

The detected 11 CpGs in all three cohorts have the potential to serve as the candidate epigenetic markers for changes in lung function during adolescence in females.

## Background

The period from childhood to adolescence is associated with rapid somatic growth and incorporates a range of gender-dependent physiological and behavioral changes, including hormonal, height and body mass index (BMI) changes, possible use of oral contraceptives, and possible initiation of nicotine use [1, 2]. This period is also significant for the development of lung function as it represents a phase of dramatic growth from childhood to adolescence to reach a maximal level of lung function in early adulthood [3–5]. Lung function growth is gender-dependent and such dependence is attributable to multiple biological determinants, including dimensional/anatomical (e.g., airway size, somatic growth, lung growth, adolescence growth spurts), immunological, and hormonal determinants such as different phases of the menstrual cycle and common hormonal and metabolic conditions [6–9].

DNA methylation (DNA-M), as a potential marker of past exposure or significant changes in life such as pubertal onset, is an epigenetic mechanism and has been shown to play an important role in human development and health. DNA-M refers to methylation of the 5′ position of the cytosine base of cytosine-phosphate-guanine dinucleotide sites (CpG sites or CpGs) in the DNA [10]. It regulates gene function through the modulation of gene expression. Imboden et al. 2019 [11] and others have demonstrated that DNA-M in whole blood is associated with lung function [12–16], risk of asthma [17], and chronic obstructive pulmonary disease (COPD) [12, 13, 15, 16]. When assessing the association of DNA-M with lung function, most previous studies have been cross-sectional with both lung function and DNA-M measured at single time points [12–16], although DNA-M at some CpGs changes over time [18–22]. In our recent genome-wide study, we identified more than 10 K CpGs where DNA-M significantly changes over the adolescence period, and at some CpGs, such changes were gender-dependent [23].

To our knowledge, at CpGs which are potentially associated with lung function parameters such as forced expiratory volume in one second (FEV_1_) and forced vital capacity (FVC), no studies have examined whether and how changes in DNA-M at those CpGs are associated with changes in lung function during adolescence. Such an investigation will improve our understanding of epigenetic mechanisms in lung function development. In addition, DNA-M changes at CpGs shown to be associated with changes in lung function have the potential to predict future lung function changes, which, in the long run, may lead to strategies for the prevention of pulmonary disease. Taken together, we hypothesized that during adolescence, changes of DNA-M at some CpGs are associated with changes in lung function. Given that changes during adolescence are gender-dependent, we examined this hypothesis separately in males and females. The study was carried out in a birth cohort located on the Isle of Wight (IOW) in the United Kingdom. To assess generalizability, the findings were further examined in two independent birth cohorts, Avon Longitudinal Study of Children and Parents Cohort (ALSPAC) in the United Kingdom and Children, Allergy, Milieu, Stockholm, Epidemiology (BAMSE) in Sweden.

## Methods

### Discovery cohort - IOW cohort

#### Study participants

The IOW cohort is a population-based birth cohort and was established in 1989 on the IOW, United Kingdom. The study was approved by the IOW Local Research Ethics Committee at recruitment initial assessments and further assessments were approved by the National Research Ethics Service, Committee South Central – Southampton B (06/Q1701/34). Informed written consent was obtained from participants or their parents before participating. The study enrolled 1456 eligible children of 1536 born between January 1989 and February 1990 (after exclusion of adoptions, infant deaths, and denial). Details of the birth cohort of 1989 have been described elsewhere [24]. Longitudinal monitoring of diseases and assessments of environmental exposures in this cohort was conducted at birth, and ages 1, 2, 4, 10, 18, and 26 years. In the present study, we focused on data collected at ages 10 (*n* = 1373) and 18 (*n* = 1313) years. In total 320 and 453 participants had both DNA-M and lung function data available at ages 10 and 18 years, respectively, including 301 participants that had data at both time points.

#### Lung function

Spirometric measurements, specifically, FVC and FEV_1_ at ages 10 (*n* = 980) and 18 (*n* = 838) years were conducted using a Koko spirometer and software with a portable desktop device (both PDS Instrumentation, Louisville, KY, USA) and the ratio of FEV_1_ over FVC (FEV_1_/FVC) was calculated. Spirometry was conducted and evaluated according to the American Thoracic Society (ATS) guidelines [25, 26]. Participants were required to be free of respiratory infection and had not taken oral steroids for two weeks. In addition, participants were instructed to abstain from any β-agonist medication for six hours and caffeine intake for at least 4 h.

#### Measuring DNA methylation (DNA-M)

Peripheral blood samples collected at ages 10 (*n* = 330) and 18 (*n* = 476) years from randomly selected subjects were used for DNA extraction via a standard salting out procedure [27]. DNA concentration was estimated by Qubit quantitation. For each sample, one microgram DNA was bisulfite-treated for cytosine to thymine conversion using the EZ 96-DNA methylation kit (Zymo Research, Irvine, CA, USA), following the manufacturer’s protocol. DNA-M was measured using HumanMethylation450K or HumanMethylationEPIC BeadChips (Illumina, Inc., SanDiego, CA, USA). Arrays were processed using a standard protocol as described elsewhere [28], with multiple identical control samples assigned to each bisulfite conversion batch to assess assay variability. DNA samples were randomly distributed on microarrays to control against batch effects. Intensities of methylated and unmethylated sites were measured.

#### Preprocessing

Probes not reaching a detection *p*-value of 10^− 16^ in at least 95% of samples were excluded. CpGs on sex chromosomes were also excluded to avoid potential bias in DNA-M as there are the parent of origin differences in methylation of paternally and maternally inherited X chromosomes [29]. DNA-M data were pre-processed using the “CPACOR” pipeline for data from both platforms [30]. DNA-M intensities were quantile normalized using the R computing package, *minfi* [31]. DNA-M β values for each CpG was calculated as a ratio of methylated (M) over the sum of methylated and unmethylated (U) probes (β = M/[c + M + U]) interpreted as the percentage of methylation [32], where c is used as a constant to prevent zero in the denominator. Principal components (PCs) inferred based on control probes were used to represent latent variables due to chip-to-chip and technical (batch) variation. Since DNA-M data were from two different platforms (450 K and EPIC), we determined the PCs based on DNA-M at shared control probes between the two platforms. The 450 K BeadChips contained 220 control probes and the EPIC BeadChips contained 204 control probes, of which 195 overlapped between the two platforms. These 195 shared probes were then used to calculate the control probe PCs, top 15 of which were used to represent latent batch factors [30].

After pre-processing, a total of 473,864 and 847,155 CpGs were available in the 450K and EPIC methylation array data, respectively, and 439,635 overlappings CpGs were identified between the two platforms. CpGs with a single nucleotide polymorphisms (SNP) overlapping the detection probe with minor allele frequency ≥ 0.7% in Caucasians (corresponding to at least 10 subjects in the IOW cohort with *n* = 1456) within 10 base pairs of the targeted CpGs were excluded due to potential bias that those SNPs brought to the measurement of DNA-M. After excluding probe SNPs, 402,714 CpGs were included in the statistical analyses.

#### Confounders

Variables potentially associated with lung function change in addition to DNA-M change in adolescents are considered to be confounders, including changes in height and BMI, age of puberty onset, smoking status, socioeconomic status (SES), exposure to pets, exposure to air pollution, education status, farm exposure, paracetamol (acetaminophen) use, and non-steroidal anti-inflammatory drugs (NSAIDs) use [33–36].

Gender information was collected by questionnaire at each follow-up. Height was measured at 10 and 18 years of age before spirometric assessment. BMI was calculated from height and weight at age 10 and 18 years. Then changes of the height and BMI were calculated from age 10 to 18 years. The minimum age of puberty onset was estimated based on the following questions about the age of initiation of different pubertal changes: growth spurt of male or female, body hair growth of male or female, skin changes of male or female, deepening voice of male, facial hair of male, breast development of female, and initiation of menstruation of female. Smoking status was defined by the questions of current and past personal smoking status at age 18 years. A composite “SES-cluster” variable that accounts for SES broadly defined was used [37]. In order to correctly classify them, family SES were clustered using: (a) British socioeconomic classes (1-6) derived from parental occupation reported at birth; (b) number of children in the index child’s bedroom (collected at age 4 years); and (c) family income at age 10 years [37]. This composite variable captures the family social class across the entire study period. Information on exposure to cats, dogs, and other animals was collected at both ages 10 and 18 years via questionnaire. Information on whether the subjects are still in education (yes/no), farm exposure (yes/no), how often health is affected by exposing to air pollution (never/ every day/ once a month/ once a week/ once a year), paracetamol use (frequency of taking paracetamol in a month) and use of NSAIDs (frequency of taking NSAIDs in a month) were collected by questionnaire at age 18 years.

### Replication cohort – the ALSPAC cohort

The Avon Longitudinal Study of Children and Parents (ALSPAC) is a population-based birth cohort study established in 1991 in Avon, United Kingdom, approximately 75 miles from the IOW. Details of the cohort were described elsewhere [38, 39]. Women residing in the South West of England who were pregnant and expecting to deliver between April 1, 1991 and December 31, 1992 were eligible to be recruited. In total, 14,541 pregnant women were eligible for the study, of those 13,761 were included with 10,321 providing DNA from blood samples. Participants were given questionnaires to gauge information regarding the mother. Written informed consent was obtained for all ALSPAC participants. Ethical approval for the study was obtained from the ALSPAC Ethics and Law Committee and the Local Research Ethics Committees. Information on environment, lifestyle, and health of the child and family was collected through annual questionnaires since the child’s birth. From age 7 years, all participants were invited to an annual research clinic, and thus exposure and other demographic data were available annually from 7 to 17 years. The follow-up cohort was composed of 13,988 children including multiple children from one family. In the replication study, we focused on ages 7 to 8 (7/8) and 15 years. Spirometry (Vitalograph 2120; Vitalograph, Maids Moreton, United Kingdom) was performed at 8 and 15 years of age according to ATS standards [26, 36], the same method as that applied in the IOW cohort. Please note that the study website contains details of all the data that is available through a fully searchable data dictionary and variable search tool (http://www.bristol.ac.uk/alspac/researchers/our-data/).

DNA-M in peripheral blood was assessed using the Infinium HumanMethylation450K BeadChip. The procedure for DNA sample preparation was comparable to that applied in the IOW cohort. DNA-M data of children at ages 7 (*n* = 966) and 15 (*n* = 966) years were available (twin participants were excluded). The pre-processing of DNA-M was performed by adjusting the batch effect, excluding CpGs with detection *p*-value ≥0.01, and excluding samples that were flagged a sex-mismatch based on X-chromosome methylation [40]. CpGs on sex chromosomes were not included in the analyses. Only fully characterized subjects with DNA-M and lung function at both ages (7/8 years and 15 years) were included in the replication study, which resulted in 691 paired samples.

### Replication cohort – the BAMSE cohort

The Swedish Children, Allergy, Milieu, Stockholm, Epidemiology (BAMSE) cohort is an unselected, population-based cohort study of children from Stockholm, Sweden. During 1994–1996, a total of 4089 children were recruited at birth from four municipalities in Stockholm County and followed during childhood. The Regional Ethical Review Board, Karolinska Institute in Stockholm, Sweden, approved the baseline study with its follow-up. A thorough description of the cohort, inclusion and enrollment criteria, and procedure of data collection have been described elsewhere [41]. Follow-up questionnaires focusing on the children’s respiratory health, allergic diseases and on various exposure factors were collected at 1, 2, 4, 8, and 16 years old after obtaining informed consent from the parents of all participating children. At ages 8 (*n* = 1838) and 16 (*n* = 2063) years, lung function testing was conducted [42]. Maximal expiratory flow volume (MEFV) tests were performed at 8 and 16 years of age using the 2200 Pulmonary Function Laboratory (Sensormedics, Anaheim, CA, USA) and Jaeger MasterScreen-IOS system (Carefusion Technologies, San Diego, CA), respectively [42, 43]. All children performed several MEFV measurements and the maximal values of FVC and FEV_1_ were extracted for the analyses. The MEFV curve that passed visual quality inspection, and the two highest FEV_1_ and FVC readings were reproducible according to ATS/ European Respiratory Society criteria [26]. FEV_1_/FVC ratios were calculated. Height was measured before lung function testing for each participant.

DNA extracted from peripheral blood samples at ages 8 and 16 years of follow up was used to measure DNA-M [44]. For each sample, 500 ng DNA underwent bisulfite treatment for cytosine to thymine conversion using the EZ 96-DNA methylation kit (Shallow; Zymo Research Corporation, Irvine, CA, USA). DNA-M was assessed using the Illumina Infinium HumanMethylation450K BeadChip (Illumina, Inc.). After data preprocessing and quality control following the standard criteria [45], DNA-M data of 464 and 267 participants were available at ages 8 and 16 years, respectively.

### Statistical analyses in the IOW cohort

To evaluate whether subjects included in the study reasonably represented those in the complete study cohort, we focused on the assessment of lung function at each age for both genders together and for each gender separately. To compare with the complete cohort, for continuous variables, including lung function, height, and BMI, one-sample t-tests were applied, and for categorical variables, including gender and smoking status, one-sample proportion tests were implemented.

Due to heteroscedasticity of DNA-M measured by β values [32], β values were logit-transformed to M values using log2 (β value/(1- β value)) [46]. Lung function measurements (FVC, FEV_1_, and FEV_1_/FVC) at each age were adjusted by height and gender by regressing lung functions on these two variables using SAS 9.4 procedure PROC GLM (SAS, Gary, N.C., USA).

In this study, we focused on lung-function-related CpGs. To achieve this goal, we first excluded CpGs which were not potentially associated with lung function. A screening package, *ttScreening* (training and testing screening, R package 3.3.2 version) [47, 48] was applied for this purpose. This method utilizes training and testing data in robust linear regressions with surrogate variables included in the regressions to adjust for unknown effects. For each lung function measure (FVC, FEV_1_, and FEV_1_/FVC), we performed the screening for each gender (males and females) at each age (10 and 18 years).

DNA-M measured in peripheral blood might be potentially influenced by cellular composition of blood samples, different batches for DNA-M measurement, and technical variation in the process of analyzing DNA samples. To adjust the impact of these factors on DNA-M, linear regressions were applied with DNA-M as the outcome variable, and cell type proportions, batch information, and top 15 principal components of the control probes were included as independent variables for age 10 and 18 years. Cell type proportions (CD4+ T cells, CD8+ T cells, natural killer cells, B cells, monocytes, neutrophils, and eosinophils) were inferred from methylation data for each sample using the R computing package *minfi* [31, 49]. After estimating the adjusted DNA-M for each age (10 and 18 years), differences in the adjusted DNA-M between ages 10 and 18 were calculated (DNA-M at age 18 – DNA-M at age 10) and included in subsequent analyses.

Finally, to explore whether the changes of DNA-M over the adolescence period from ages 10 to 18 years were associated with the change in lung function, a linear regression model was fitted for each lung function measure, stratified by gender. Changes in height- and gender-adjusted lung function from 10 to 18 years of age were treated as the outcome variable, and changes of the adjusted DNA-M at each CpG that passed screening were used as an independent variable and potential confounders as described above were included in the model. In all analyses, *p*-values were considered significant at a level of 0.05.

### Replication analyses

CpGs identified in the IOW cohort were further tested in both the ALSPAC and BAMSE cohorts. Comparable analytical methods were applied except for the availability of some covariates. In ALSPAC, pet exposure, exposure to pollution, paracetamol use, and non-steroidal anti-inflammatory drugs use were not available, and in BAMSE, minimum age of puberty onset, pet exposure, exposure to pollution, and paracetamol use were not included in the final model.

### Pathway analysis

For CpGs that showed consistent directions of association in the ALSPAC and BAMSE cohorts, the nearest gene was identified based on Illumina array manifest file and SNIPPER (https://csg.sph.umich.edu/ boehnke/snipper/) version 1.2. Bioinformatic assessment of the genes was conducted using the online bioinformatics tool ToppFun, available in the ToppGene Suite [50]. Multiple testing was adjusted by controlling the false discovery rate (FDR) of 0.05.

## Results

### Results from the IOW cohort

In total, 320 participants at age 10 years and 453 at age 18 years were included in the analyses for screening in the IOW cohort with available DNA-M and lung function data (Table 1). The mean values of FVC, FEV_1_, FEV_1_/FVC, height, and BMI for subjects in the present study were not significantly different from participants of the whole cohort with lung function at ages 10 (*n* = 980) and 18 (*n* = 838) years (Table 1) and for males and females separately with lung function at ages 10 (males = 488, females = 492) and 18 (males = 395, females = 443) (Table 2). Proportions of subjects who smoke or formerly smoked were also comparable to those in the complete cohort (Tables 1 and 2). One exception is that at age 10 years, a higher proportion of males were included in the present study compared to the whole cohort (Table 1).

**Table 1:** Characteristics of subjects with available methylation data with their lung function of the IOW cohort

IOW cohort						
	Sub cohort at age 10: Participants with lung function *Mean ± SD*	Study sample at age 10: Participants with lung function and DNA-M *Mean ± SD*	*P-* values	Sub cohort at age 18: Participants with lung function *Mean ± SD*	Study sample at age 18: Participants with lung function and DNA-M *Mean ± SD*	*P-*values
**Factors**	***n*** **= 980**	***n*** **= 320**		***n*** **= 838**	**453**	
*Lung Function parameters*						
*FEV*_*1*_*(L)*	2.03 ***±*** 0.30	2.04 ***±*** 0.30	0.456	4.01 ± 0.78	4.05 ± 0.76	0.226
*FVC (L)*	2.30 ***±*** 0.34	2.30 ***±*** 0.34	0.694	4.61 ± 0.93	4.66 ± 0.91	0.283
*FEV*_*1*_*/FVC*	0.89 ***±*** 0.06	0.89 ***±*** 0.05	0.162	0.87 ± 0.07	0.87 ± 0.07	0.269
*Height (cm)*	138.92 ***±*** 6.18	138.06 ***±*** 6.22	0.685	170.88 ± 9.17	170.92 ± 9.08	0.925
*BMI*	18.16 ***±*** 3.01	18.08 ***±*** 2.99	0.649	23.21 ± 4.33	23.30 ± 4.26	0.639
	***n*(%)**	***n*(%)**		***n*(%)**	***n*(%)**	
*Gender*						
*Male*	488 (49.8)	183 (57.19)	**0.010**	395 (47.14)	212 (46.80)	0.932
*Female*	492 (50.2)	137 (42.81)		443 (52.86)	241 (53.20)	
*Smoking*^*a*^						
*Non- smoker*	–	–	–	462 (55.13)	248 (54.75)	0.913
*Current smoker*	–	–		204 (24.34)	107 (23.62)	
*Past smoker*	–	–		159 (18.97)	93 (20.53)	
*Missing*	–	–		13 (1.55)	5 (1.10)	

^*a*^Active smoking at age 10 years in the IOW Cohort was not identified

**Table 2:** Characteristics of subjects with methylation data and lung function of IOW cohort, stratified by gender

IOW cohort						
	Sub cohort at age 10: Participants with lung function *Mean ± SD*	Study sample at age 10: Participants with lung function and DNA-M *Mean ± SD*	*P-* values	Sub cohort at age 18: Participants with lung function *Mean ± SD*	Study sample at age 18: Participants with lung function and DNA-M *Mean ± SD*	*P-*values
**Factors**	***n*** **= 488**	***n*** **= 183**		***n*** **= 395**	***n*** **= 212**	
**Males**						
*FEV*_*1*_*(L)*	2.05 ***±*** 0.30	2.06 ***±*** 0.29	0.477	4.62 ± 0.62	4.64 ± 0.62	0.621
*FVC (L)*	2.35 ***±*** 0.34	2.36 ***±*** 0.33	0.813	5.35 ± 0.72	5.35 ± 0.73	0.930
*FEV*_*1*_*/FVC*	0.88 ***±*** 0.06	0.88 ***±*** 0.06	0.651	0.87 ± 0.07	0.87 ± 0.07	0.904
*Height (cm)*	139.00 ***±*** 5.90	138.94 ***±*** 5.95	0.893	177.83 ± 6.65	177.56 ± 6.87	0.568
*BMI*	17.57 ***±*** 2.52	17.65 ***±*** 2.54	0.665	22.51 ± 3.72	22.64 ± 3.72	0.609
	***n*(%)**	***n*(%)**		***n*(%)**	***n*(%)**	
*Smoking*^*a*^						
*Non- smoker*	–	–	–	222 (56.20)	119 (56.13)	0.813
*Current smoker*	–	–		93 (23.54)	46 (21.70)	
*Past smoker*	–	–		72 (18.23)	44 (20.75)	
*Missing*	–	–		8 (2.03)	3 (1.42)	
**Females**	***n*** **= 492**	***n*** **= 137**		***n*** **= 443**	***n*** **= 241**	
*FEV*_*1*_*(L)*	2.00 ***±*** 0.29	2.01 ***±*** 0.29	0.832	3.51 ± 0.45	3.53 ± 0.43	0.340
*FVC (L)*	2.23 ***±*** 0.33	2.24 ***±*** 0.34	0.657	4.03 ± 0.53	4.04 ± 0.51	0.737
*FEV*_*1*_*/FVC*	0.90 ***±*** 0.06	0.90 ***±*** 0.05	0.337	0.88 ± 0.07	0.87 ± 0.07	0.563
*Height (cm)*	139.02 ***±*** 6.43	139.22 ***±*** 6.58	0.719	164.68 ± 6.17	165.08 ± 6.37	0.331
*BMI*	18.74 ***±*** 3.34	18.66 ***±*** 3.41	0.789	23.84 ± 4.72	23.89 ± 4.61	0.870
	***n*(%)**	***n*(%)**		***n*(%)**	***n*(%)**	
*Smoking*^*a*^						
*Non- smoker*	–	–	–	240 (54.18)	129 (53.53)	0.979
*Current smoker*	–	–		111 (25.06)	61 (25.31)	
*Past smoker*	–	–		87 (19.64)	49 (20.33)	
*Missing*	–	–		5 (1.13)	2 (0.83)	

^*a*^Active smoking at age 10 years in the IOW Cohort was not identified

**Fig. 1:**
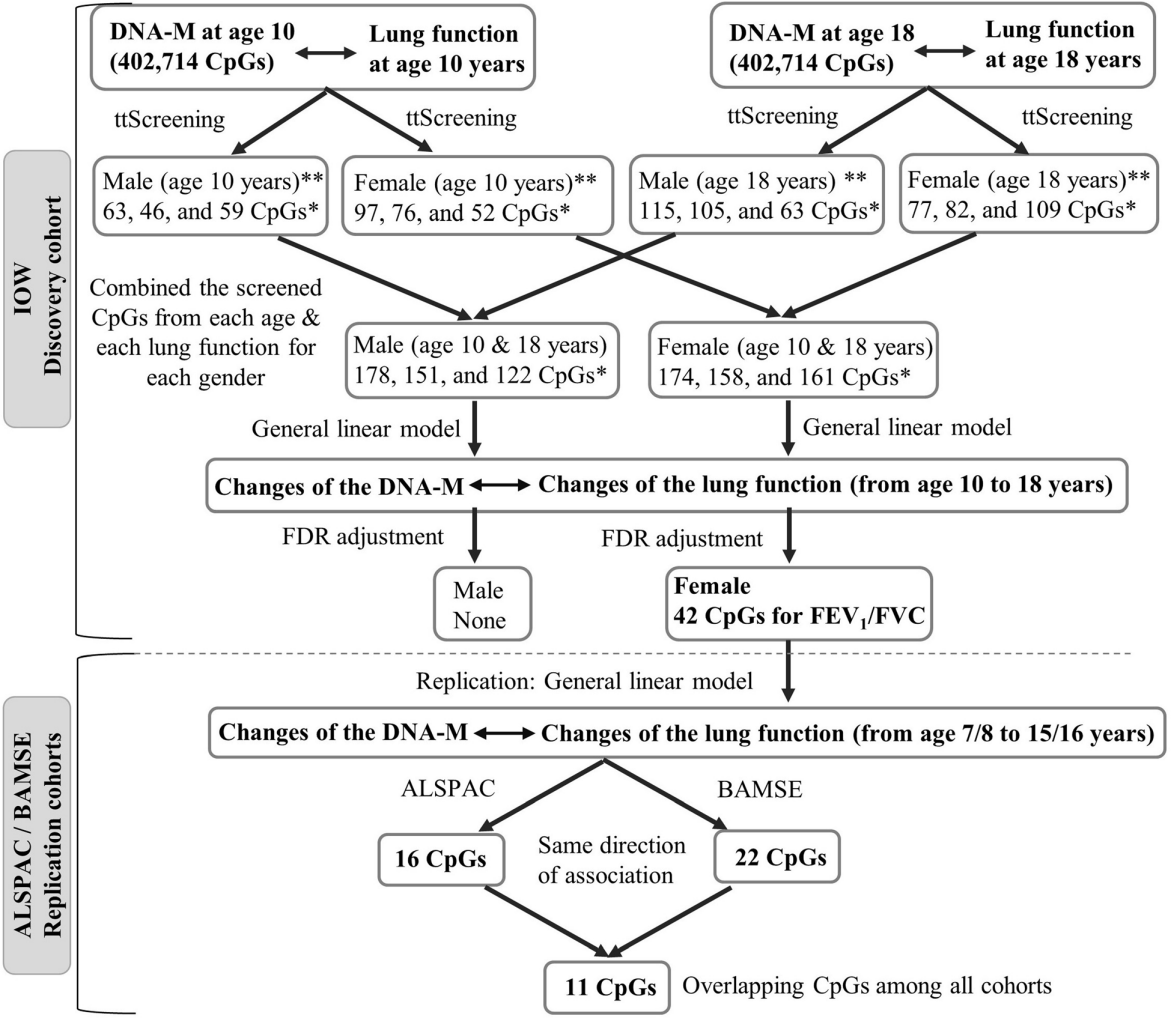
Flow chart of statistical analyses and the number of CpGs after each analysis. Note: 1) *Number of significant CpGs were mentioned in an order for FVC, FEV_1_, and FEV_1_/ FVC changes respectively. 2) **At age 10 years, for males, between FVC and FEV_1_, and between FEV_1_ and FEV_1_/ FVC, 8 and 3 CpGs are overlapped, respectively; for females, between FVC and FEV_1_, 21 CpGs are overlapped in the screening. 3) At age 18 years, for males, between FVC and FEV_1_, and between FEV_1_ and FEV_1_ / FVC, 8 and 1 CpGs are overlapped, respectively; for females, between FVC and FEV_1_, between FEV_1_ and FEV_1_/ FVC, and between FVC and FEV_1_/ FVC, 9, 1, and 2 CpGs are overlapped, respectively, in the screening

To identify candidate CpGs potentially associated with lung function at ages 10 and 18 years, we applied *ttScreening* to the 402,714 CpGs in each gender. Three lung function parameters were considered in the screening process, FVC, FEV_1_, and FEV_1_/FVC. At age 10 years, across all the three lung function parameters, in total 361 distinct CpGs passed screening (157 CpGs for males and 204 CpGs for females), and at age 18 years, 530 distinct CpGs passed screening (274 CpGs for males and 256 CpGs for females). The break-down of the numbers of CpGs that passed screening for each lung function parameter was given in Fig. 1. Combining the CpGs that passed the screening at either time point for each gender and each lung function measurement, in males 431 distinct CpGs (178 CpGs for FVC, 151 for FEV_1_, and 122 for FEV_1_/FVC) and in females 460 distinct CpGs (174 CpGs for FVC, 158 for FEV_1_, and 161 FEV_1_/FVC) were included in the subsequent analyses. There were no common CpGs between the 431 and 460 CpGs identified in males and females.

Linear regression models were applied to assess the association of change in DNA-M at each of the screened CpG with the change of each lung function parameter (FVC, FEV_1_, and FEV_1_/FVC) for males (*n* = 169) and females (*n* = 132) separately. For females, after adjusting for multiple testing by controlling the FDR of 0.05, 42 CpGs showed statistically significant association with FEV_1_/FVC change, but for FEV_1_ and FVC, we did not identify any statistically significant CpGs. At these 42 CpGs, a larger increase in DNA-M was associated with a larger decrease in FEV_1_/FVC in females. From childhood to adolescence, generally FEV_1_/FVC is constant or falls linearly with age because FVC has a proportionately greater increase than FEV_1_ [51], which supports our findings. For males, no CpG survived multiple testing for any of the three lung function parameters. The 42 CpGs identified in females in the IOW cohort were further tested in the ALSPAC and BAMSE cohorts.

### Results from the ALSPAC cohort

**Fig. 2:**
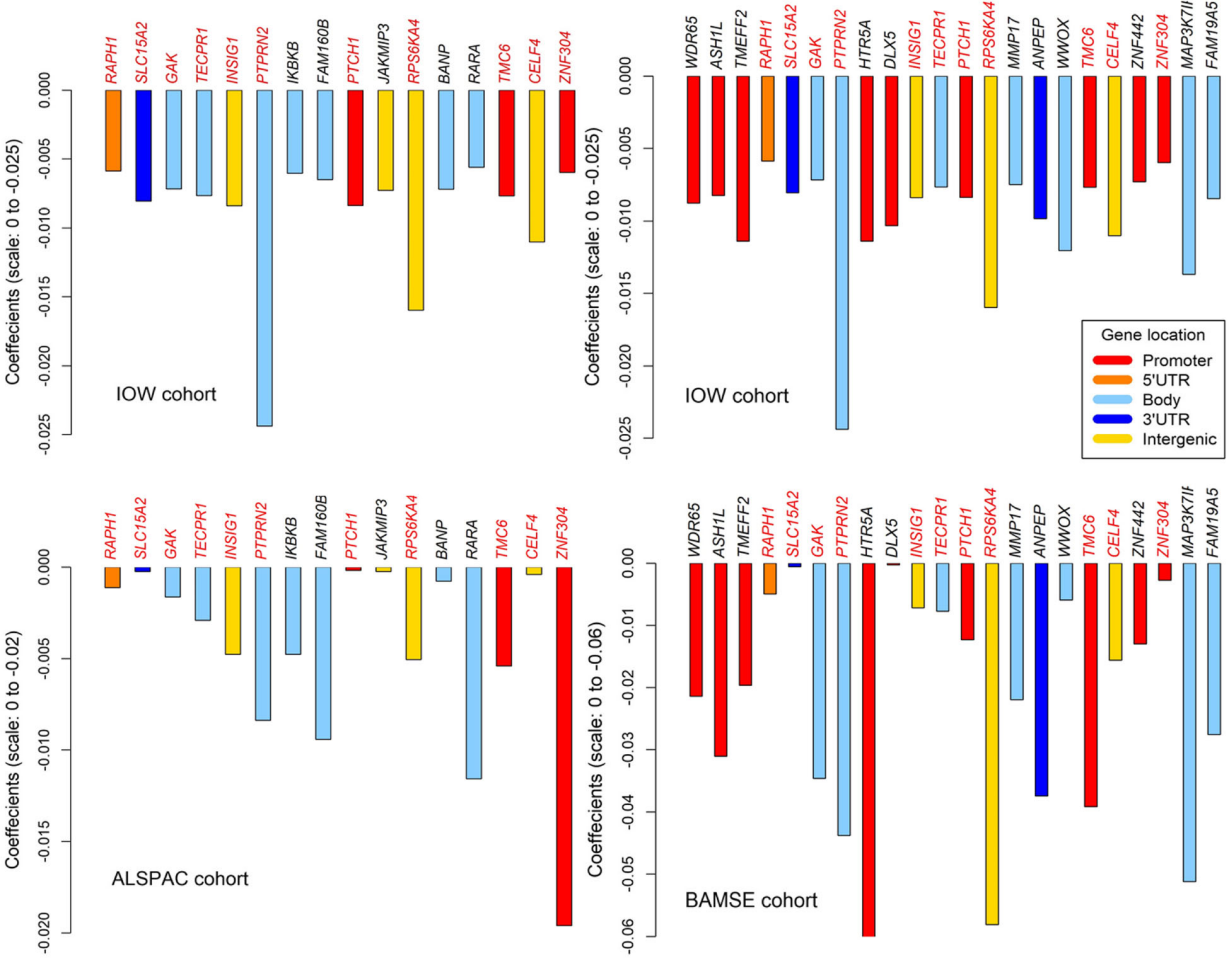
Barplots of coefficients of IOW-ALSPAC and IOW-BAMSE consistent CpGs with their mapped genes in females. Note: The coefficients were shown for the association of DNA-M changes with changes in lung function (FEV_1_/FVC) in females adolescence. Mapped genes of the CpGs showing consistent associations between the IOW and ALSPAC cohorts (left panels) and between the IOW and BAMSE cohorts (right panels) were included. Gene names overlapped among the three cohorts were given in red font

In total, 345 female (*n* = 935) participants in the ALSPAC had FEV_1_/FVC measurements and DNA-M measurements at both 7/8 years and 15 years old. Of the 42 CpGs examined, DNA-M changes at 16 CpGs (Table 3) showed consistent associations with FEV_1_/FVC changes (in terms of regression coefficients) compared to those observed in the IOW cohort (Fig. 2, Table 3), although not statistically significant at the 0.05 level. These 16 CpGs were noted as IOW-ALSPAC consistent CpGs. The complete results of this analysis were included in Additional file 1: Table S1.

**Table 3:** CpGs showing consistent associations in females between the IOW and replication cohorts, ALSPAC and BAMSE

CpG Name	Chr.	Gene name	Location	IOW cohort			ALSPAC-cohort		BAMSE cohort	
				Coeff.	*P*_*Raw*_-value	*P*_*FDR*_ -value	Coeff.	*P*-value	Coeff.	*P*-value
cg08095278	1	*ASH1L*	TSS1500	−0.008	0.0031	0.0218	–	–	−0.031	0.339
cg13342625	1	*WDR65*	TSS200	−0.009	0.0016	0.0187	–	–	−0.021	0.663
cg02288301	2	*TMEFF2*	TSS1500	−0.011	0.0021	0.0201	–	–	−0.020	0.549
**cg08366885**	**2**	***RAPH1***	**5’UTR**	**−0.006**	**0.0092**	**0.0381**	**−0.001**	**0.889**	**−0.005**	**0.758**
**cg16710348**	**3**	***SLC15A2***	**3’UTR**	**−0.008**	**0.0114**	**0.0436**	**−0.0002**	**0.975**	**−0.001**	**0.980**
**cg09839318**	**4**	***GAK***	**Body**	**−0.007**	**0.0084**	**0.0364**	**−0.002**	**0.762**	**−0.035**	**0.163**
cg00930455	7	*DLX5*	TSS1500	−0.010	0.0055	0.0299	–	**–**	−0.0003	0.990
**cg04132649**	**7**	***TECPR1***	**Body**	**−0.008**	**0.0071**	**0.0345**	**−0.003**	**0.573**	**−0.008**	**0.770**
cg14552568	7	*HTR5A*	Intergenic	−0.011	0.0012	0.0187	–	–	−0.091	**0.006**
**cg15575249**	**7**	***INSIG1***	**Intergenic**	**−0.008**	**0.0014**	**0.0187**	**−0.005**	**0.523**	**−0.007**	**0.826**
**cg21584493**	**7**	***PTPRN2***	**Body**	**−0.024**	**0.0033**	**0.0218**	**−0.008**	**0.287**	**−0.044**	**0.369**
cg09573852	8	*IKBKB*	Body	−0.006	0.0054	0.0299	−0.005	0.399	–	–
cg23188819	8	*FAM160B2*	Body	−0.007	0.0030	0.0218	−0.009	0.422	–	–
**cg14319249**	**9**	***PTCH1***	**TSS200**	**−0.008**	**0.0021**	**0.0201**	**−0.0002**	**0.987**	**−0.012**	**0.684**
cg09033333	10	*JAKMIP3*	Intergenic	−0.007	0.0035	0.0223	−0.0002	0.977	–	–
**cg01082111**	**11**	***RPS6KA4***	**Intergenic**	**−0.016**	**0.0002**	**0.0092**	**−0.005**	**0.551**	**−0.058**	**0.051**
cg07427606	12	*MMP17*	Body	−0.007	0.0031	0.0218	–	–	−0.022	0.412
cg05312779	15	*ANPEP*	3’UTR	−0.010	0.0062	0.0315	–	–	−0.037	0.220
cg04575609*	16	*BANP*	Body	−0.007	0.0021	0.0201	−0.001	0.884	–	–
cg04933438	16	*WWOX*	Body	−0.012	0.0002	0.0092	–	–	−0.006	0.849
cg11316510	17	*RARA*	Body	−0.006	0.0100	0.0403	−0.012	0.290	–	–
**cg11493223**	**17**	***TMC6***	**TSS200**	**−0.008**	**0.0027**	**0.0217**	**−0.005**	**0.703**	**−0.039**	**0.186**
**cg13206530**	**18**	***CELF4***	**Intergenic**	**−0.011**	**0.0026**	**0.0217**	**−0.0004**	**0.961**	**−0.016**	**0.621**
cg00850039	19	*ZNF442*	TSS200	−0.007	0.0056	0.0299	–	–	−0.013	0.710
**cg10157975**	**19**	***ZNF304***	**TSS1500**	**−0.006**	**0.0063**	**0.0315**	**−0.020**	**0.256**	**−0.003**	**0.909**
cg10027934	22	*MAP 3K7IP1*	Body	−0.014	0.0012	0.0187	–	–	−0.051	0.081
cg27652464	22	*FAM19A5*	Body	−0.008	0.0022	0.0201	–	–	−0.028	0.219

Note: 1) Regression coefficients were for the associations of changes in DNA-M with FEV_1_/FVC changes in females

2) CpGs with the genes’ names in **bold** font were overlapped across all the three cohorts (IOW-ALSPAC-BAMSE consistent CpGs)

3) In BAMSE cohort, DNA-M of *cg04575609 was excluded at the time of quality control and was not available for the replication analysis

4) *Chr*. chromosome number, *Coeff*. coefficients

### Results from the BAMSE cohort

In the BAMSE cohort, 48 female participants had lung function and DNA-M data at ages 8 and 16 years, and DNA-M at 41 of the 42 CpGs were available in these 48 females. At 22 of the 41 CpGs, the associations of DNA-M changes with changes in FEV_1_/FVC were consistent with the findings in the IOW cohort, with one CpG showing statistical significance at 0.05 level (cg14552568) and two CpGs approached significance (cg01082111 and cg10027934, *p*-value < 0.1). These 22 CpGs were noted as IOW-BAMSE consistent CpGs, of which 11 of these IOW-BAMSE consistent CpGs were among the 16 IOW-ALSPAC consistent CpGs. These 11 CpGs were further noted as IOW-ALSPAC-BAMSE consistent CpGs.

### Findings of the biological pathway analysis

Genes to which CpGs showed consistent results in either of the two cohorts (ALSPAC and BAMSE) in terms of the direction of associations mapped to were included in the pathway analyses. The 16 IOW-ALSPAC consistent CpGs were mapped to 16 genes, and 22 genes were identified for the 22 IOW-BAMSE consistent CpGs (Table 3). The selected 16 and 22 genes were further investigated to discover the functional enrichment in the biological process by using the bioinformatics tool ToppFun.

In total, eight biological processes were identified from the FDR adjusted *p*-value of 0.05 (Table 4). Eight genes, *CELF4, INSIG1, PTCH1, RPS6KA4, ZNF304, RARA, IKBKB,* and *BANP* to which the IOW-ALSPAC consistent CpGs were mapped, were involved in most of the eight biological processes. The same biological processes were found that involved genes *CELF4, INSIG1, PTCH1, RPS6KA4, ZNF304, DLX5, WWOX,* and *ASH1L* corresponding to the IOW-BAMSE consistent CpGs, although they did not survive multiple testing.

**Table 4:** Biological processes were identified from the mapped genes based on the IOW-ALSPAC consistent CpGs

Name of the Biological process	*P*_*Raw*_*-*value	*P*_*FDR*_ -value	Hit in Query List
Positive regulation of RNA metabolic process	3.39E-05	2.93E-02	***CELF4, INSIG1, PTCH1, RPS6KA4, ZNF304,****RARA, IKBKB, BANP*
Positive regulation of nucleobase-containing compound metabolic process	9.30E-05	2.93E-02	***CELF4****,****INSIG1****,****PTCH1****,****RPS6KA4****,****ZNF304****, RARA, IKBKB, BANP*
Positive regulation of gene expression	1.16E-04	2.93E-02	***CELF4, INSIG1, PTCH1, RPS6KA4, ZNF304****, RARA, IKBKB, BANP*
Interleukin-1-mediated signaling pathway	1.33E-04	2.93E-02	***RPS6KA4****, IKBKB*
Positive regulation of nitrogen compound metabolic process	1.42E-04	2.93E-02	***CELF4, INSIG1, PTCH1, RPS6KA4, ZNF304****, RARA, IKBKB, BANP*
Positive regulation of nucleic acid-templated transcription	2.30E-04	3.19E-02	***INSIG1, PTCH1, RPS6KA4, ZNF304****, RARA, IKBKB, BANP*
Positive regulation of transcription, DNA-templated	2.30E-04	3.19E-02	***INSIG1, PTCH1, RPS6KA4, ZNF304****, RARA, IKBKB, BANP*
Positive regulation of RNA biosynthetic process	2.47E-04	3.19E-02	***INSIG1, PTCH1, RPS6KA4, ZNF304****, RARA, IKBKB, BANP*

Note: The same biological processes were involved in the BAMSE cohort based on the CpGs identified BAMSE, although they did not survive multiple testing after controlling for FDR of 0.05

Genes which were formatted in **bold**, involved in the biological processes in both ALSPAC and BMASE cohorts

## Discussion

Limited studies have focused on longitudinal lung function and DNA-M measurements during adolescence, an important period of life that significantly contributes to lung function development [36, 43]. The present study is the first genome-scale exploration of the association of changes of DNA-M with changes in lung function during adolescence, stratified by gender. We showed that DNA-M changes in 11 CpGs were associated with changes in FEV_1_/FVC in females in adolescence, based on findings from the IOW cohort and two independent cohorts. Such associations were not identified in males. It is important to mention that, the final results focused on the direction of associations rather than statistical significance as non-equivalence of statistical significance and clinical significance has been recognized [52, 53]. We suggest that in replication studies agreement in clinical significance should be more important than statistical significance, although it will be most desirable when an agreement is reached in clinical significance accompanied by statistical significance.

Among the genes involved in the identified biological processes based on the findings in both ALSPAC and BAMSE cohorts, genes *INSIG1*, *PTCH1*, and *PTPRN2* have been shown in a range of studies for their involvement in lung development, lung function, and inflammatory airway diseases such as asthma and COPD [54–60], although most findings were not specifically linked to adolescence. Gene *INSIG1* allied with cg15575249 encodes the protein, insulin induced gene 1, which plays a significant role in regulating lipogenesis in alveolar types 2 cells consistent with the roles of sterol regulatory element-binding protein (SREBP)/ sterol cleavage-activating protein in lung lipid synthetic pathways [54]. *INSIG1* is primarily involved in epithelial development and surfactant physiology during the perinatal period [55]. The findings in our study further emphasize its importance in the change of lung function in adolescence.

Gene *PTCH1* allied with cg14319249 encodes a member of the patched family of proteins that functions as a receptor and a component of the hedgehog (Hh) signaling pathway [56–58]. The Hh signaling pathway is crucial in embryonic lung development processes, including the morphogenesis of lung and regulating the interaction between epithelial and mesenchymal cell populations in the airway and alveolar compartments [56–58]. Sonic Hh (one type of Hh signaling) is active in adult lung function [57, 58], but to our knowledge, its relation to lung function changes in adolescence has not been examined before. The link of *PTCH1* with FEV_1_/FVC was also established in a genome-wide association study meta-analysis by the CHARGE consortium [59]. CpGs cg21584493 is mapped to gene *PTPRN2*. In a recent study, differentially methylated region (DMR) annotated to *PTPRN2* genes was identified for the association with lung function and asthma in children [60]. Findings in our study on these genes (*INSIG1*, *PTCH1*, and *PTPRN2*) further emphasizes their epigenetic contribution to the changes in lung function in adolescence.

CpGs cg11316510 and cg09573852 on genes *RARA* (retinoic acid receptor alpha) and *IKBKB* (Inhibitor of Nuclear Factor Kappa B Kinase), respectively, were among the IOW-ALSPAC consistent CpGs but not on the list of IOW-BAMSE consistent CpGs. Their significant involvement in lung function, as well as lung function development and pulmonary diseases such as asthma and COPD indicated the potential importance of these two CpGs and their mapped genes [11, 61–71]. *RARA* is the predominant isotype of the retinoic acid receptor (RAR) identified in alveolar type II epithelial cells and components of the retinoic acid signaling pathway [63–68]. The retinoic acid signaling pathway plays important roles in lung development and alveolarization, and to regulate surfactant protein B gene expression in pulmonary epithelial cells. Adolescence is a period accompanied by significant lung function development and the functionality of this pathway supports the findings in our study. One of our recent studies also showed an epigenetic association of *RARA* with FEV_1_/FVC [11].

*IKBKB* is an enzyme complex that forms part of the nuclear factor-kappa B signaling pathway, which has been considered the master regulator of immune responses and demonstrated to play a cardinal role in allergic airways diseases [69–71]. In addition, gene *IKBKB* was required for the IL17-dependent signaling that was associated with neutrophilia and pulmonary inflammation [72].

It is worth noting that the genes discussed above were based on the findings in females in our study. For CpGs located on those genes, no statistically significant associations were shown in males. The identified unique 11 CpGs in three population-based cohorts thus have the potential to serve as epigenetic markers related to lung function development during adolescence in females, but not in males. The absence of such epigenetic associations in males led us to postulate the possibility of either different underlying epigenetic mechanisms in each gender in the regulation of gene activity, or that these CpGs are biomarkers of female physiology and/or exposures that influence lung function growth in adolescence. Thus, our findings may help to explain the various gender-associated health conditions related to lung function development in adolescence, such as gender reversal of asthma incidence in males and females.

There are some limitations of this study. Firstly, DNA-M measurements were made in peripheral blood leukocytes and provide no insight into epigenetic changes in structural cells of the airway. Secondly, concurrent instead of time-lagged modeling was applied to assess the association of DNA-M changes with lung function changes for each gender. In this context, we were not able to examine the potential of changes in DNA-M at the identified CpGs to predict lung function changes. In the IOW cohort, the analyses were based on data collected at ages 10 and 18 years representing pre- and post-adolescence. In the two replication cohorts, however, the corresponding ages were 7–8 years and 15 years for ALSPAC and 8 and 16 years for BAMSE. It is likely that many participants at age 15/16 years were still in the transition period or even just started puberty. This possibility accompanied by potentially significant changes in DNA-M during adolescence [23] might explain the non-replication of some CpGs identified in the IOW cohort. Other potential contributors to this non-replication may include some covariates being unavailable in the replication cohorts as well as variable characteristics unique to each cohort. On the other hand, the 11 CpGs showing consistent associations across all the three cohorts certainly deserve further assessment of their generalizability, as well as on the potential of predicting lung function changes.

## Conclusions

This epigenetic study represents an integrated strategy to understand lung function changes in males and females during adolescence. We identified 11 CpGs as potential markers for lung function development, which are applicable to females only. Findings from the study provide insight into the role of epigenetics in gender-dependent lung function development during this critical period of life and thus providing a strong foundation to evaluate gender reversal of asthma from male to female in adolescence period. In subsequent studies, the detected 11 CpGs could serve as candidate epigenetic markers to predict changes in lung function during adolescence.

## Supplementary information

**Additional file 1 Table S1**. List of CpGs (k = 42) at which changes of DNA-M were significantly associated with changes of FEV_1_/FVC in females in IOW cohort and examined among the females in ALSPAC and BAMSE cohorts.

## Data Availability

The datasets used and/or analyzed during the current study are available from the corresponding author on reasonable request.

## Abbreviations

ATS: American Thoracic Society
ALSPAC: Avon Longitudinal Study of Children and Parents
BAMSE: Children, Allergy, Milieu, Stockholm, Epidemiology
BMI: Body mass index
CpGs: Cytosine-phosphate-guanine dinucleotide site or sites
COPD: Chronic obstructive pulmonary disease
DNA-M: DNA methylation
FVC: Forced vital capacity
FEV_1_: Forced expiratory volume in one second
FDR: False discovery rate
Hh signal: Hedgehog signal
IOW: Isle of Wight
PCs: Principal components
*ttScreening*: Training and testing screening

## References

[1] Guthikonda K, Zhang H, Nolan VG, Soto-Ramirez N, Ziyab AH, Ewart S (2014). Oral contraceptives modify the effect of GATA3 polymorphisms on the risk of asthma at the age of 18 years via DNA methylation. Clin Epigenetics.

[2] Yousefi M, Karmaus W, Zhang H, Ewart S, Arshad H, Holloway J (2013). The methylation of the LEPR/LEPROT genotype at the promoter and body regions influence concentrations of leptin in girls and BMI at age 18 years if their mother smoked during pregnancy. Int J Mol Epidemiol Genet.

[3] Piccioni P, Tassinari R, Carosso A, Carena C, Bugiani M, Bono R (2015). Lung function changes from childhood to adolescence: a seven-year follow-up study. BMC Pulm Med.

[4] Mahmoud O, Granell R, Tilling K, Minelli C, Garcia-Aymerich J, Holloway JW, Custovic A, Jarvis D, Sterne J, Henderson J. Association of height growth in puberty with lung function: a longitudinal study. Am J Respir Crit Care Med. 2018;198(12):1539-48. 10.1164/rccm.201802-0274OC PMC6298631 29995435

[5] Berry CE, Billheimer D, Jenkins IC, Lu ZJ, Stern DA, Gerald LB, Carr TF, Guerra S, Morgan WJ, Wright AL, et al. A distinct low lung function trajectory from childhood to the fourth decade of life. 2016;194(5):607–12. 10.1164/rccm.201604-0753OC PMC5027213 27585385

[6] Becklake MR, Kauffmann F (1999). Gender differences in airway behaviour over the human life span. Thorax.

[7] Carey MA, Card JW, Voltz JW, Arbes SJ, Germolec DR, Korach KS (2007). It's all about sex: gender, lung development and lung disease. Trends Endocrinol Metab.

[8] LoMauro A, Aliverti A (2018). Sex differences in respiratory function. Breathe (Sheff).

[9] Almqvist C, Worm M, Leynaert B (2008). Working group of GALENWPG. Impact of gender on asthma in childhood and adolescence: a GA2LEN review. Allergy.

[10] Moore LD, Le T, Fan G (2013). DNA methylation and its basic function. Neuropsychopharmacology.

[11] Imboden M, Wielscher M, Rezwan FI, Amaral André FS, Schaffner E, Jeong A, Beckmeyer-Borowko A, Harris SE, Starr JM, Deary Ian J, et al. Deary Ian J et al. Epigenome-wide association study of lung function level and its change. Eur Respir J. 2019;54(1):1900457. 10.1183/13993003.00457-2019 PMC6610463 31073081

[12] Qiu W, Baccarelli A, Carey VJ, Boutaoui N, Bacherman H, Klanderman B (2012). Variable DNA methylation is associated with chronic obstructive pulmonary disease and lung function. Am J Respir Crit Care Med.

[13] Lepeule J, Baccarelli A, Motta V, Cantone L, Litonjua AA, Sparrow D (2012). Gene promoter methylation is associated with lung function in the elderly: the normative aging study. Epigenetics..

[14] Lange NE, Sordillo J, Tarantini L, Bollati V, Sparrow D, Vokonas P, Zanobetti A, Schwartz J, Baccarelli A, Litonjua AA, et al. Alu and LINE-1 methylation and lung function in the normative ageing study. BMJ Open. 2012;2(5):e001231. 10.1136/bmjopen-2012-001231 PMC3488751 23075571

[15] Busch R, Qiu W, Lasky-Su J, Morrow J, Criner G, DeMeo D (2016). Differential DNA methylation marks and gene comethylation of COPD in African-Americans with COPD exacerbations. Respir Res.

[16] Lee MK, Hong Y, Kim SY, Kim WJ, London SJ (2017). Epigenome-wide association study of chronic obstructive pulmonary disease and lung function in Koreans. Epigenomics.

[17] Zhang H, Tong X, Holloway JW, Rezwan FI, Lockett GA, Patil V (2014). The interplay of DNA methylation over time with Th2 pathway genetic variants on asthma risk and temporal asthma transition. Clin Epigenetics.

[18] Florath I, Butterbach K, Muller H, Bewerunge-Hudler M, Brenner H (2014). Cross-sectional and longitudinal changes in DNA methylation with age: an epigenome-wide analysis revealing over 60 novel age-associated CpG sites. Hum Mol Genet.

[19] Wang D, Liu X, Zhou Y, Xie H, Hong X, Tsai HJ (2012). Individual variation and longitudinal pattern of genome-wide DNA methylation from birth to the first two years of life. Epigenetics.

[20] Madrigano J, Baccarelli AA, Mittleman MA, Sparrow D, Vokonas PS, Tarantini L (2012). Aging and epigenetics: longitudinal changes in gene-specific DNA methylation. Epigenetics.

[21] Xu C-J, Bonder MJ, Söderhäll C, Bustamante M, Baïz N, Gehring U (2017). The emerging landscape of dynamic DNA methylation in early childhood. BMC Genomics.

[22] Acevedo N, Reinius LE, Vitezic M, Fortino V, Söderhäll C, Honkanen H (2015). Age-associated DNA methylation changes in immune genes, histone modifiers and chromatin remodeling factors within 5 years after birth in human blood leukocytes. Clin Epigenetics.

[23] Han L, Zhang H, Kaushal A, Rezwan FI, Karmaus W, Henderson AJ, et al. Assessing DNA methylation changes pre- and post-adolescence and pubertal exposures via a longitudinal genome-scale study. Clinical Epigenetics. 2019;Minor revision and potential acceptable. 10.1186/s13148-019-0780-4 PMC6888960 31791392

[24] Arshad SH, Holloway JW, Karmaus W, Zhang H, Ewart S, Mansfield L (2018). Cohort Profile: The Isle Of Wight Whole Population Birth Cohort (IOWBC). Int J Epidemiol.

[25] Crapo R (2000). Guidelines for methacholine and exercise challenge testing-1999. This official statement of the American Thoracic Society was adopted by the ATS Board of directors, July 1999. Am J Respir Crit Care Med.

[26] Miller MR, Hankinson J, Brusasco V, Burgos F, Casaburi R, Coates A (2005). Standardisation of spirometry. Eur Respir J.

[27] McClelland M, Hanish J, Nelson M, Patel Y (1988). KGB: a single buffer for all restriction endonucleases. Nucleic Acids Res.

[28] Bibikova M, Fan J-B (2009). GoldenGate® assay for DNA methylation profiling.

[29] Golden LC, Itoh Y, Itoh N, Iyengar S, Coit P, Salama Y (2019). Parent-of-origin differences in DNA methylation of X chromosome genes in T lymphocytes. Proc Natl Acad Sci.

[30] Lehne B, Drong AW, Loh M, Zhang W, Scott WR, Tan ST (2015). A coherent approach for analysis of the Illumina HumanMethylation450 BeadChip improves data quality and performance in epigenome-wide association studies. Genome Biol.

[31] Aryee MJ, Jaffe AE, Corrada-Bravo H, Ladd-Acosta C, Feinberg AP, Hansen KD (2014). Minfi: a flexible and comprehensive bioconductor package for the analysis of Infinium DNA methylation microarrays. Bioinformatics..

[32] Du P, Feng G, Huang S, Kibbe WA, Lin S. Analyze Illumina Infinium methylation microarray data. 2012.

[33] Hollams EM, de Klerk NH, Holt PG, Sly PD (2014). Persistent effects of maternal smoking during pregnancy on lung function and asthma in adolescents. Am J Respir Crit Care Med.

[34] Patil VK, Holloway JW, Zhang H, Soto-Ramirez N, Ewart S, Arshad SH (2013). Interaction of prenatal maternal smoking, interleukin 13 genetic variants and DNA methylation influencing airflow and airway reactivity. Clin Epigenetics.

[35] Weiss ST (2010). Lung function and airway diseases. Nat Genet.

[36] Sonnenschein-van der Voort AM, Howe LD, Granell R, Duijts L, Sterne JA, Tilling K (2015). Influence of childhood growth on asthma and lung function in adolescence. J Allergy Clin Immunol.

[37] Ogbuanu IU, Karmaus W, Arshad SH, Kurukulaaratchy RJ, Ewart S (2009). Effect of breastfeeding duration on lung function at age 10 years: a prospective birth cohort study. Thorax.

[38] Boyd A, Golding J, Macleod J, Lawlor DA, Fraser A, Henderson J (2013). Cohort profile: the 'children of the 90s'--the index offspring of the Avon longitudinal study of parents and children. Int J Epidemiol.

[39] Fraser A, Macdonald-Wallis C, Tilling K, Boyd A, Golding J, Davey Smith G (2013). Cohort profile: the Avon longitudinal study of parents and children: ALSPAC mothers cohort. Int J Epidemiol.

[40] Relton CL, Gaunt T, McArdle W, Ho K, Duggirala A, Shihab H (2015). Data resource profile: accessible resource for integrated Epigenomic studies (ARIES). Int J Epidemiol.

[41] Hallberg J, Ballardini N, Almqvist C, Westman M, van Hage M, Lilja G (2019). Impact of IgE sensitization and rhinitis on inflammatory biomarkers and lung function in adolescents with and without asthma. Pediatr Allergy Immunol.

[42] Schultz ES, Hallberg J, Andersson N, Thacher JD, Pershagen G, Bellander T (2018). Early life determinants of lung function change from childhood to adolescence. Respir Med.

[43] Schultz ES, Gruzieva O, Bellander T, Bottai M, Hallberg J, Kull I (2012). Traffic-related air pollution and lung function in children at 8 years of age: a birth cohort study. Am J Respir Crit Care Med.

[44] Gref A, Merid SK, Gruzieva O, Ballereau S, Becker A, Bellander T (2017). Genome-wide interaction analysis of air pollution exposure and childhood asthma with functional follow-up. Am J Respir Crit Care Med.

[45] Gruzieva O, Merid SK, Melén E. An update on epigenetics and childhood respiratory diseases. Paediatr Respir Rev. 2014;15:348-54. 10.1016/j.prrv.2014.07.003 25151612

[46] Du P, Zhang X, Huang CC, Jafari N, Kibbe WA, Hou L (2010). Comparison of Beta-value and M-value methods for quantifying methylation levels by microarray analysis. BMC Bioinformatics.

[47] Li X, Hawkins GA, Ampleford EJ, Moore WC, Li H, Hastie AT (2013). Genome-wide association study identifies TH1 pathway genes associated with lung function in asthmatic patients. J Allergy Clin Immunol.

[48] Ray MA, Tong X, Lockett GA, Zhang H, Karmaus WJ (2016). An efficient approach to screening Epigenome-wide data. Biomed Res Int.

[49] Houseman EA, Accomando WP, Koestler DC, Christensen BC, Marsit CJ, Nelson HH (2012). DNA methylation arrays as surrogate measures of cell mixture distribution. BMC Bioinformatics..

[50] Chen J, Aronow BJ, Jegga AG (2009). Disease candidate gene identification and prioritization using protein interaction networks. BMC Bioinformatics.

[51] Karmaus W, Mukherjee N, Janjanam VD, Chen S, Zhang H, Roberts G (2019). Distinctive lung function trajectories from age 10 to 26 years in men and women and associated early life risk factors - a birth cohort study. Respir Res.

[52] Altman DG, Bland JM (1995). Statistics notes: absence of evidence is not evidence of absence. BMJ.

[53] Altman DG, Gore SM, Gardner MJ, Pocock SJ (1983). Statistical guidelines for contributors to medical journals. British Med J (Clin Res Ed).

[54] Insig1 Regulates SREBP Mediated Lipogenesis In Alveolar Type 2 Cells. C61 Gene regulation during development and in injury. p. A4953-A.

[55] Bridges JP, Schehr A, Wang Y, Huo L, Besnard V, Ikegami M (2014). Epithelial SCAP/INSIG/SREBP signaling regulates multiple biological processes during perinatal lung maturation. PloS One.

[56] Li X, Howard TD, Moore WC, Ampleford EJ, Li H, Busse WW (2011). Importance of hedgehog interacting protein and other lung function genes in asthma. J Allergy Clin Immunol.

[57] Kugler MC, Joyner AL, Loomis CA, Munger JS (2015). Sonic hedgehog signaling in the lung. From development to disease. Am J Respir Cell Mol Biol.

[58] Tam A, Hughes M, McNagny KM, Obeidat M, Hackett TL, Leung JM (2019). Hedgehog signaling in the airway epithelium of patients with chronic obstructive pulmonary disease. Sci Rep.

[59] Hancock DB, Eijgelsheim M, Wilk JB, Gharib SA, Loehr LR, Marciante KD (2010). Meta-analyses of genome-wide association studies identify multiple loci associated with pulmonary function. Nat Genet.

[60] den Dekker HT, Burrows K, Felix JF, Salas LA, Nedeljkovic I, Yao J (2019). Newborn DNA-methylation, childhood lung function, and the risks of asthma and COPD across the life course. Eur Respir J.

[61] Na H, Lim H, Choi G, Kim BK, Kim SH, Chang YS (2018). Concomitant suppression of TH2 and TH17 cell responses in allergic asthma by targeting retinoic acid receptor-related orphan receptor gammat. J Allergy Clin Immunol.

[62] Xu L, Sun WJ, Jia AJ, Qiu LL, Xiao B, Mu L (2018). MBD2 regulates differentiation and function of Th17 cells in neutrophils- dominant asthma via HIF-1α. J Inflamm (Lond).

[63] Yang L, Naltner A, Yan C (2003). Overexpression of dominant negative retinoic acid receptor alpha causes alveolar abnormality in transgenic neonatal lungs. Endocrinology.

[64] Desai TJ, Chen F, Lu J, Qian J, Niederreither K, Dolle P (2006). Distinct roles for retinoic acid receptors alpha and beta in early lung morphogenesis. Dev Biol.

[65] Wongtrakool C, Malpel S, Gorenstein J, Sedita J, Ramirez MI, Underhill TM (2003). Down-regulation of retinoic acid receptor alpha signaling is required for sacculation and type I cell formation in the developing lung. J Biol Chem.

[66] Manoli SE, Smith LA, Vyhlidal CA, An CH, Porrata Y, Cardoso WV (2012). Maternal smoking and the retinoid pathway in the developing lung. Respir Res.

[67] Yang L, Lian X, Cowen A, Xu H, Du H, Yan C (2004). Synergy between signal transducer and activator of transcription 3 and retinoic acid receptor-alpha in regulation of the surfactant protein B gene in the lung. Mol Endocrinol (Baltimore, Md).

[68] Mendelsohn C, Lohnes D, Decimo D, Lufkin T, LeMeur M, Chambon P (1994). Function of the retinoic acid receptors (RARs) during development (II). Multiple abnormalities at various stages of organogenesis in RAR double mutants. Dev (Cambridge, England).

[69] Janssen-Heininger YM, Poynter ME, Aesif SW, Pantano C, Ather JL, Reynaert NL (2009). Nuclear factor κB, airway epithelium, and asthma: avenues for redox control. Proc Am Thorac Soc.

[70] Pannicke U, Baumann B, Fuchs S, Henneke P, Rensing-Ehl A, Rizzi M (2013). Deficiency of innate and acquired immunity caused by an IKBKB mutation. N Engl J Med.

[71] Edwards MR, Bartlett NW, Clarke D, Birrell M, Belvisi M, Johnston SL (2009). Targeting the NF-κB pathway in asthma and chronic obstructive pulmonary disease. Pharmacol Ther.

[72] Esposito S, Ierardi V, Daleno C, Scala A, Terranova L, Tagliabue C (2014). Genetic polymorphisms and risk of recurrent wheezing in pediatric age. BMC Pulmonary Medicine.

