# Supplementary material for: Changes of DNA methylation are associated with changes in lung function during adolescence
Source: Respir Res. 2020 Apr 7;21:80. doi: 10.1186/s12931-020-01342-y (PMC7140357; doi:10.1186/s12931-020-01342-y)
Supplement: Supplementary file 1 — Additional file 1 Table S1. List of CpGs (k = 42) at which changes of DNA-M were significantly associated with changes of FEV1/FVC in females in IOW cohort and examined among the females in ALSPAC and BAMSE cohorts. [file 12931_2020_1342_MOESM1_ESM.docx]

**Table S1: List of CpGs (k=42) at which changes of DNA-M were significantly associated with changes of FEV_1_/FVC in females in IOW cohort and examined among the females in ALSPAC and BAMSE cohorts.**

| **CpG Name** | **Chromosome Number** | **Gene name** | **Location** | **IOW cohort** | | | **ALSPAC cohort** | | **BAMSE cohort** | |
| --- | --- | --- | --- | --- | --- | --- | --- | --- | --- | --- |
|  |  |  |  | **Coeff.** | ***P_Raw value_*** | ***P_FDR-adjusted value_*** | **Coeff.** | ***P* value** | **Coeff.** | **P value** |
| cg08095278 | 1 | *ASH1L* | TSS1500 | -0.008 | 0.0031 | 0.022 | 0.014 | 0.085 | -0.031 | 0.339 |
| cg13342625 | 1 | *WDR65* | TSS200 | -0.009 | 0.0016 | 0.019 | 0.006 | 0.499 | -0.021 | 0.663 |
| cg02288301 | 2 | *TMEFF2* | TSS1500 | -0.011 | 0.0021 | 0.020 | 0.004 | 0.532 | -0.020 | 0.549 |
| **cg08366885** | **2** | ***RAPH1*** | **5'UTR** | **-0.006** | **0.0092** | **0.038** | **-0.001** | **0.889** | **-0.005** | **0.758** |
| cg19515245 | 2 | *Not found* | Intergenic | -0.012 | 0.0015 | 0.019 | 0.009 | 0.090 | 0.015 | 0.517 |
| **cg16710348** | **3** | ***SLC15A2*** | **3'UTR** | **-0.008** | **0.0114** | **0.044** | **-0.0002** | **0.975** | **-0.001** | **0.980** |
| **cg09839318** | **4** | ***GAK*** | **Body** | **-0.007** | **0.0084** | **0.036** | **-0.002** | **0.763** | **-0.035** | **0.163** |
| cg19290091 | 4 | *STIM2* | Intergenic | -0.012 | 0.0009 | 0.019 | 0.002 | 0.803 | 0.016 | 0.562 |
| cg21237481 | 5 | *DUSP1* | Intergenic | -0.008 | 0.008 | 0.036 | 0.018 | **0.030** | 0.021 | 0.431 |
| cg00930455 | 7 | *DLX5* | TSS1500 | -0.010 | 0.0055 | 0.030 | 0.025 | **0.003** | 0.000 | 0.990 |
| **cg04132649** | **7** | ***TECPR1*** | **Body** | **-0.008** | **0.0071** | **0.035** | **-0.003** | **0.573** | **-0.008** | **0.770** |
| cg14552568 | 7 | *HTR5A* | Intergenic | -0.011 | 0.0012 | 0.019 | 0.004 | 0.444 | -0.091 | **0.006** |
| **cg15575249** | **7** | ***INSIG1*** | **Intergenic** | **-0.008** | **0.0014** | **0.019** | **-0.005** | **0.530** | **-0.007** | **0.826** |
| **cg21584493** | **7** | ***PTPRN2*** | **Body** | **-0.024** | **0.0033** | **0.022** | **-0.008** | **0.287** | **-0.044** | **0.369** |
| cg21617762 | 7 | *MNX1* | Intergenic | -0.008 | 0.0056 | 0.030 | 0.001 | 0.947 | 0.004 | 0.873 |
| cg09573852 | 8 | *IKBKB* | Body | -0.006 | 0.0054 | 0.030 | -0.005 | 0.399 | 0.003 | 0.893 |
| cg23188819 | 8 | *FAM160B2* | Body | -0.007 | 0.003 | 0.022 | -0.009 | 0.422 | 0.023 | 0.382 |
| **cg14319249** | **9** | ***PTCH1*** | **TSS200** | **-0.008** | **0.0021** | **0.020** | **-0.0002** | **0.987** | **-0.012** | **0.684** |
| cg09033333 | 10 | *JAKMIP3* | Intergenic | -0.007 | 0.0035 | 0.022 | -0.0002 | 0.977 | 0.024 | 0.495 |
| cg09978996 | 10 | *TET1* | 5'UTR | -0.011 | 0.0005 | 0.014 | 0.015 | 0.122 | 0.012 | 0.681 |
| **cg01082111** | **11** | ***RPS6KA4*** | **Intergenic** | **-0.016** | **0.0002** | **0.009** | **-0.005** | **0.551** | **-0.058** | **0.051** |
| cg01436586 | 11 | *HRAS* | Body | -0.006 | 0.011 | 0.043 | 0.007 | 0.339 | 0.014 | 0.552 |
| cg09035529 | 11 | *SLC22A11* | Body | -0.008 | 0.0091 | 0.038 | 0.001 | 0.885 | 0.027 | 0.316 |
| cg07427606 | 12 | *MMP17* | Body | -0.007 | 0.0031 | 0.022 | 0.004 | 0.519 | -0.022 | 0.412 |
| cg18996808 | 14 | *INF2* | Body | -0.015 | 0.0015 | 0.019 | 0.008 | 0.320 | 0.045 | 0.182 |
| cg05312779 | 15 | *ANPEP* | 3'UTR | -0.010 | 0.0062 | 0.032 | 0.005 | 0.665 | -0.037 | 0.220 |
| cg09155362 | 15 | *RYR3* | Intergenic | -0.010 | 0.0003 | 0.009 | 0.012 | 0.057 | 0.016 | 0.564 |
| cg04575609* | 16 | *BANP* | Body | -0.007 | 0.0021 | 0.020 | -0.001 | 0.884 | - | - |
| cg04933438 | 16 | *WWOX* | Body | -0.012 | 0.0002 | 0.009 | 0.002 | 0.837 | -0.006 | 0.849 |
| cg09488452 | 16 | *PARN* | 5'UTR | -0.015 | 0.0003 | 0.009 | 0.008 | 0.310 | 0.019 | 0.417 |
| cg16739679 | 16 | *ZNF720* | TSS1500 | -0.011 | 0.0011 | 0.019 | 0.010 | 0.388 | 0.013 | 0.722 |
| cg11316510 | 17 | *RARA* | Body | -0.006 | 0.01 | 0.040 | -0.012 | 0.290 | 0.013 | 0.615 |
| **cg11493223** | **17** | ***TMC6*** | **TSS200** | **-0.008** | **0.0027** | **0.022** | **-0.005** | **0.703** | **-0.039** | **0.186** |
| cg12222323 | 17 | *ERBB2* | TSS200 | -0.011 | 0.0051 | 0.030 | 0.001 | 0.894 | 0.008 | 0.702 |
| cg14501061 | 17 | *ALDOC* | TSS200 | -0.006 | 0.0079 | 0.036 | 0.006 | 0.407 | 0.017 | 0.556 |
| **cg13206530** | **18** | ***CELF4*** | **Intergenic** | **-0.011** | **0.0026** | **0.022** | **-0.0004** | **0.963** | **-0.016** | **0.621** |
| cg00850039 | 19 | *ZNF442* | TSS200 | -0.007 | 0.0056 | 0.030 | 0.010 | 0.414 | -0.013 | 0.710 |
| cg00926680 | 19 | *OAZ1* | TSS200 | -0.008 | 0.0076 | 0.036 | 0.003 | 0.678 | 0.018 | 0.572 |
| **cg10157975** | **19** | ***ZNF304*** | **TSS1500** | **-0.006** | **0.0063** | **0.032** | **-0.020** | **0.256** | **-0.003** | **0.909** |
| cg21967668 | 19 | *FGF21* | TSS200 | -0.013 | 0.0003 | 0.009 | 0.001 | 0.876 | 0.002 | 0.944 |
| cg10027934 | 22 | *MAP3K7IP1* | Body | -0.014 | 0.0012 | 0.019 | 0.008 | 0.187 | -0.051 | 0.081 |
| cg27652464 | 22 | *FAM19A5* | Body | -0.008 | 0.0022 | 0.020 | 0.005 | 0.410 | -0.028 | 0.219 |

The coefficients that go in the same direction in both cohorts were in bold font. The *p* values <0.05 in replication cohorts were in red font.

* In BAMSE cohort, DNA-M of cg04575609 was excluded at the time of quality control and was not available for the replication analysis.
